# Hybrid quadrupole plasmon induced spectrally pure ultraviolet emission from a single AgNPs@ZnO:Ga microwire based heterojunction diode†

**DOI:** 10.1039/c9na00777f

**Published:** 2020-02-24

**Authors:** Xiangbo Zhou, Mingming Jiang, Yuting Wu, Kunjie Ma, Yang Liu, Peng Wan, Caixia Kan, Daning Shi

**Affiliations:** College of Science, Nanjing University of Aeronautics and Astronautics No. 29 Jiangjun Road Nanjing 210016 China mmjiang@nuaa.edu.cn cxkan@nuaa.edu.cn shi@nuaa.edu.cn; Key Laboratory for Intelligent Nano Materials and Devices (MOE), Nanjing University of Aeronautics and Astronautics Nanjing 210016 China

## Abstract

Ultraviolet light-emitting materials and devices with high-efficiency are required for many applications. One promising way to enhance the ultraviolet luminescence efficiency is by incorporating plasmonic nanostructures. However, a large energy mismatch between the plasmons and the light emitters greatly limits the direct realization of light enhancement. In this work, a single Ga-doped ZnO microwire prepared with large-sized Ag nanoparticle (the diameter *d* ∼ 200 nm) deposition (AgNPs@ZnO:Ga MW) was utilized to construct a high-performance heterojunction diode, with p-GaN serving as the hole injection layer. In addition to enhanced optical output, the emission spectra also revealed that typical near-band-edge (NBE) emission with higher wavelength stability centered around 378.0 nm was achieved, accompanied by narrowing of the spectral linewidth to around 10 nm. Thus, the interfacial and p-GaN emissions were successfully suppressed. The spectral profile of the emission spectra of the heterojunction diodes precisely matched the photoluminescence spectrum of the single ZnO:Ga MW, which indicates that the single ZnO:Ga MW can act as the active region for the radiative recombination of electrons and holes in the diode operation. In the emission mechanism, hybrid quadrupole plasmons induce the generation of hot electrons, which are then injected into the conduction band of the neighboring ZnO:Ga and are responsible for the NBE-type emission of the single MW based heterojunction diode. This novel emission enhancement and modulation principle can aid in the design and development of new types of luminescent materials and devices with high-efficiency, spectral stability and spectral purity.

## Abstract 2

In this work, a single Ga-doped ZnO microwire prepared with large-sized Ag nanoparticle deposition (AgNPs@ZnO:Ga MW) was utilized to construct a high-performance heterojunction diode, with p-GaN serving as the hole injection layer.

## Graphical abstract

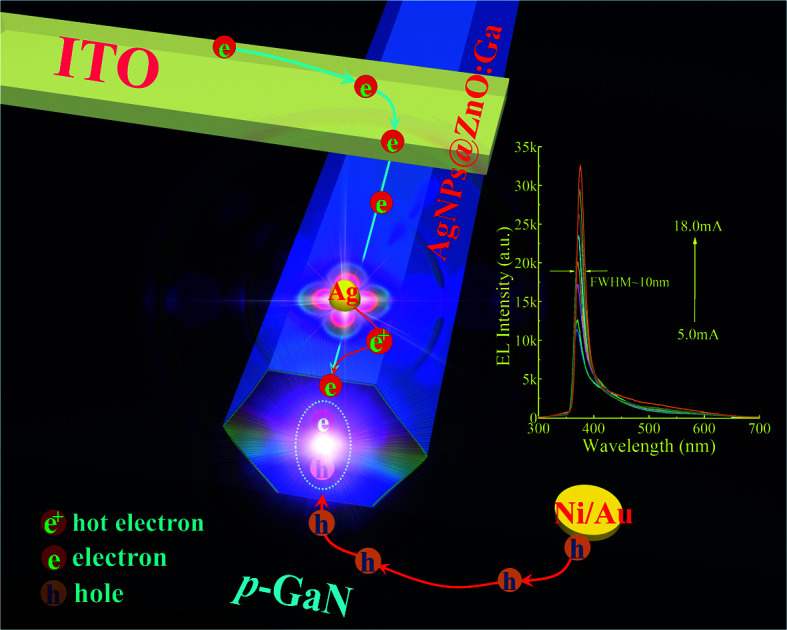

## 1 Introduction

Due to its outstanding piezoelectric characteristics, excellent electronic transport and optical features, superior optical gain behavior, biocompatibility, chemical stability and so on, ZnO (wide bandgap of 3.37 eV, and large exciton binding energy of 60 meV) has been widely used in versatile applications, for instance high-efficiency photonic devices and short-wavelength optoelectronic devices (*e.g.*, ultraviolet light-emitting diodes, laser diodes, and ultraviolet-blind photodetectors).^1–5^ Within the comprehensive investigations of ZnO, there is increasing interest in nano/microstructures, which have already demonstrated their incomparable utility as field effect transistors, optically and electrically pumped lasers, photodetectors, batteries, and chemical and biological sensors.^6–8^ For the past few years, great efforts have also been devoted to preparing low-dimensional ZnO nano/microstructures, such as by metal–organic chemical vapor deposition, thermal evaporation, chemical vapor transport, laser ablation, and so on.^9–13^ Specifically, it is well-known that interface roughness is an important factor in influencing the optical and electrical performances of low-dimensional photoelectronic devices. Thus, balancing the surface defects and dopants in nano/microstructures without having a detrimental effect on their optical and electrical behavior is a challenge. Furthermore, due to surface defects and low carrier injection efficiency, nano/microstructures exhibit broadband emissions and inherently low external efficiency, which severely reduce their ability to meet scientific and commercial demands.^14–16^

When coupled with light at specific photon energies, collective oscillations of conduction electrons in metallic nanostructures can be excited. The spectral properties of these excitations, called plasmons, have attracted widespread scientific and technological interest. The incorporation of metal nanostructures, predominantly in their elemental forms, can be employed to significantly modulate the optical and optoelectronic performances of semiconductors.^17–21^ By incorporating noble metal nanostructures, such as Ag nanoparticles, the localized surface plasmon resonance effect has been considered to be an efficient strategy to enhance the luminous efficiency of light-emitting materials and devices, with ZnO micro/nanostructures serving as active layers.^22–24^ Due to the small sizes of the plasmonic nanostructures, the optical response of the spherical nanostructures embedded in the dielectric materials exhibits dipole surface plasmon resonance modes, which result in strong attenuation of incident light. Nevertheless, owing to serious metallic losses arising from interband electronic transitions, metal plasmons are trapped in the visible spectral band.^25–29^ These metallic losses are detrimental to the performance of plasmon mediated optoelectronic devices, seriously limiting the feasibility of many plasmonic applications. Taking Ag nanospheres into account, the dipole plasmons usually appear in the visible and near-infrared spectral bands, thus, it is difficult to achieve effective emission enhancement of ultraviolet emission devices in the short wavelength spectral band.^30–32^ Compared with traditional dipole plasmons, high-order plasmonic modes can also be excited in the shorter wavelength spectral region by means of adjusting the gap distance in metal nanostructure aggregates, as well as increasing the size. Therefore, the excitation of high-order plasmonic modes, as well as their application in ultraviolet optoelectronic devices, has not only attracted attention in the current investigation, but also in more in-depth studies of the scientific problems.^33–37^

In this work, individual Ga-doped ZnO microwires covered by large-sized Ag nanoparticles (*d* ∼ 200 nm) (AgNPs@ZnO:Ga MWs) were prepared. Heterojunction diodes composed of a single MW were fabricated, with p-GaN serving as the hole injection layer. In addition to significant enhancement of the ultraviolet emission, the emission spectra of the single AgNPs@ZnO:Ga MW based heterojunction light-emitting diodes showed that the dominant emission peak wavelength was centered around 378.0 nm, and that the spectral linewidth was reduced to around 10 nm. Specifically, the interfacial and p-GaN emissions were suppressed effectively. The NBE-type ultraviolet emissions of ZnO:Ga that dominated the EL spectra can be assigned to the deposited large-sized Ag nanoparticles. To further explore the emission modulation, the plasmonic characteristics of the deposited Ag nanoparticles were investigated. Due to the mismatch between the hybrid quadrupole plasmons and the ZnO:Ga excitons, it was found that the excited hybrid quadrupole plasmons in the ultraviolet spectral region could decay nonradiatively, resulting in the generation of hot electrons, which were then injected into the heterojunction diode. Therefore, the deposited large-sized Ag nanoparticles play an important role in modulating the emission features of the ultraviolet light-emitting diodes.

## 2 Experimental section

### The preparation of individual AgNPs@ZnO:Ga MWs

Individual ZnO:Ga MWs with hexagonal cross-section were successfully synthesized *via* a chemical vapor deposition (CVD) method.^24,38–40^ In the synthesis procedure, by employing high-purity Ga_2_O_3_ (99.999%) as an efficient Ga incorporation source, a premixed high-purity powder of ZnO (99.99%), Ga_2_O_3_ and graphite (C) (99.99%) with the weight ratio of 10 : 1 : 11 was used as the precursor source material.^39,41^ A corundum boat was used as a container, with a Si substrate placed on the precursor mixture to collect the products. To make sure the as-synthesized MWs exhibited high crystal quality and excellent electrical conduction, the growth temperature of the furnace chamber was maintained at 1150 °C; meanwhile, a mixed carrier gas composed of high-purity argon (Ar: 99.99%, 180 sccm) and high-purity oxygen (O_2_: 99.99%, 15 sccm), serving as the protecting gas and the reaction gas, respectively, was introduced into the furnace chamber. Finally, individual ZnO:Ga MWs could be collected around the Si substrate.

A single MW was selected and then transferred onto a quartz substrate. Indium (In) particles were employed as the electrodes and were fixed on the MW. Afterwards, Ag quasiparticle nanofilms were deposited on the MW using the magnetron sputtering technique.^23,25,38^ Thus, a single ZnO:Ga MW decorated with Ag quasiparticle nanofilms (Ag@ZnO:Ga) was fabricated. When the applied bias exceeded a certain value, bright and visible incandescent-type emission was observed, with the emission regions located towards the center of the wire. The emission features were analogous to a tungsten filament lamp.^10,38,39^ Interestingly, the Ag quasiparticle nanofilms could be annealed into physically isolated Ag nanoparticles located in the emission regions due to the Joule heating effect. Moreover, by increasing the sputtering time, the size and gap distance of the prepared Ag nanoparticle aggregates could also be modulated. Therefore, a single ZnO:Ga MW covered with Ag nanoparticle decoration (AgNPs@ZnO:Ga) was successfully prepared.^23,25,38^

### Fabrication of single MW based heterojunction diode

A heterojunction light-emitting diode composed of a single MW was constructed, with p-GaN serving as the hole injection layer. After being cleaned thoroughly, MgO was deposited on the GaN substrate using the molecular beam epitaxy technique, to serve as an insulating layer. A Ni/Au electrode with a size of 0.5 mm and a thickness of 50 nm was deposited on the GaN *via* an electron-beam evaporation system. Afterwards, the single MW was transferred onto the GaN substrate, accompanied by an In particle fixed to the MW on the MgO layer. Thereby, a single MW based heterojunction diode was fabricated.^6,23,40,42,43^

### Analysis instruments

Single ZnO:Ga MWs, Ag nanostructures, and MWs covered by Ag nanostructures were characterized using scanning electron microscopy (SEM). The current–voltage (*I*–*V*) characteristics of the single MW based metal–MW–metal structure and the single MW based heterojunction diode were investigated using a Keysight semiconductor device analyzer (B1500A). The photoluminescence (PL) was measured with a LABRAM-UV Jobin Yvon spectrometer using a He–Cd laser (325 nm) as the excitation source. The emitted photons from the electrically biased single MW based heterojunction diode were collected using a PIXIS-1024BR-CCD detection system. Optical characterization of the light emission of the heterojunction diode was also carried out *via* an optical microscope camera.

## 3 Results and discussion

**Fig. 1:**
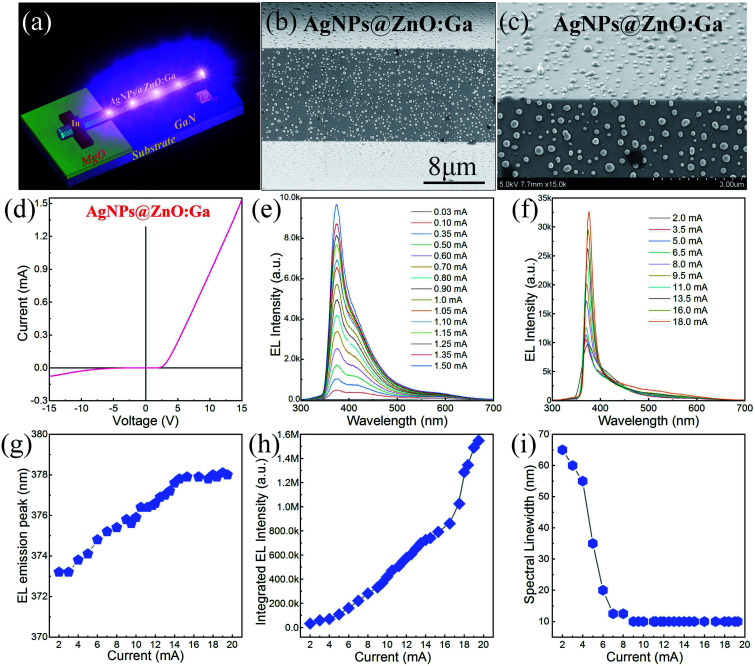
EL emission characteristics of a single AgNPs@ZnO:Ga MW based heterojunction diode: (a) schematic diagram of ultraviolet emission from a heterojunction diode composed of n-AgNPs@ZnO:Ga MW/p-GaN. (b) SEM image of a single AgNPs@ZnO:Ga MW (sputtering time: 300 s). (c) Magnified SEM image of Ag nanoparticles, with the average diameter of the nanoparticles *d* ∼ 200 nm. (d) *I*–*V* characteristics of the n-AgNPs@ZnO:Ga MW/p-GaN heterojunction light-emitting device. (e) Room-temperature EL spectra of the n-AgNPs@ZnO:Ga MW/p-GaN heterojunction diode under low forward injection current ranging from 0.03 to 1.50 mA. (f) The EL spectra collected for the single MW based heterojunction diode on further increasing the injection current from 2.0 to 18.0 mA. (g) The dominant emission peak wavelengths as a function of the injection current. (h) Integrated EL emission intensities as a function of the injection current. (i) The spectral linewidth narrowing tendency on increasing the forward injection current.

During the growth procedure of individual MWs, the Ga vapor generated by carbothermal reduction of the Ga_2_O_3_ high-purity powder can be dissolved into the excess Zn vapor. On incorporation with residual O_2_ in the furnace chamber, individual Ga-doped ZnO MWs could be gradually formed around the Si substrate.^24,38–40^ A single ZnO:Ga MW was selected to construct an incandescent-type lamp filament source, with the emission region located towards the center of the wire. By incorporating Ag quasiparticle nanofilms (the sputtering time: 300 s), a single Ag@ZnO:Ga MW based incandescent-type emitter was also fabricated. When the applied bias exceeded a certain value, bright and visible emission could be observed, with the emission regions still located towards the center. Moreover, due to the Joule heating effect, the Ag quasiparticle nanofilms could be annealed into physically isolated Ag nanoparticles in the emission regions.^23,24,38,39^ Thus, the segment of the MW covered by isolated Ag nanoparticles (AgNPs@ZnO:Ga MW) was selected to fabricate heterostructured light-emitting devices. A schematic diagram is provided to explain the emission characteristics of an electrically biased single AgNPs@ZnO:Ga MW based heterostructured light emission device at room temperature, as depicted in Fig. 1(a). In the fabrication procedure of the single AgNPs@ZnO:Ga MW based heterostructured light-emitting devices, p-GaN served as the hole injection layer and was employed as the substrate. A single AgNPs@ZnO:Ga MW was characterized, as indicated in Fig. 1(b). This showed that physically isolated Ag nanoparticles with random distribution were successfully deposited on the MW. A magnified SEM image of the Ag nanoparticles is also shown in Fig. 1(c), with average diameter ∼ 200 nm. This demonstrated that Ag nanoparticles with random distribution on the ZnO:Ga MW could be acquired.^23,38^

As described in the experimental section, a single AgNPs@ZnO:Ga MW was selected as the luminescent material to construct a heterostructured emission device. Fig. 1(d) exhibits the *I*–*V* curve, indicating typical rectifying characteristics. Thus, a heterojunction diode composed of n-AgNPs@ZnO:Ga MW/p-GaN was constructed, with the turn-on voltage estimated to be about 3.4 V. By applying a forward bias at room temperature, ultraviolet emission signals were recorded by using a PIXIS camera system. The collected EL spectra under low forward injection current ranging from 0.03 to 1.5 mA are illustrated in Fig. 1(e). It exhibited prominent ultraviolet near-band-edge (NBE) emission with the dominant emission peak wavelength centered around 372.0 nm; meanwhile, broad interfacial emission covering the range from 400 to 500 nm was also observed, exhibiting broad sub-bandgap luminescence centered around 425.0 nm. On further increasing the injection current from 2.0 to 18.0 mA, Fig. 1(f) shows that NBE-type emission dominated the EL spectra, accompanied by a reduction in the spectral linewidth to around 10 nm. A little red shift of the dominant emission peak wavelength to around 378.0 nm occurred. In particular, the interfacial emission was completely suppressed. Therefore, typical NBE emission with higher wavelength stability centered around 378.0 nm was realized by the single AgNPs@ZnO:Ga MW based heterojunction diode.^6,40,44,45^

To investigate the NBE-type emission characteristics of the single AgNPs@ZnO:Ga MW based heterojunction diodes, a plot of the dominant emission peak wavelength *versus* the injection current is depicted in Fig. 1(g). On increasing the injection current in the range from 2.0 to 18.0 mA, the emission peak wavelength exhibited a little red shift from 372.0 to 378.0 nm in the ultraviolet spectral band. The integrated emission intensity was calculated as a function of the injection current, as shown in Fig. 1(h). It exhibited a superlinear tendency, indicating the co-existence of radiative and nonradiative processes. To investigate the nonlinear behavior, the light intensity was also fitted using the exponential formula of log–log scale: *L*_iem_ ∝ *I*^*m*^, where *L*_iem_ is defined as the integrated emission intensity, with *I* being the injection current, and *m* being employed to define the nonradiative and radiative modes of the light output. When the diode was operated under low injection current, a superlinear relationship was obtained. The *m* of ∼1.23 suggested that radiative recombination increased at a faster rate than nonradiative recombination. On further increasing the injection current beyond 5.0 mA, the *m* was estimated to be about 1.8, showing that radiative recombination generally made up the major part of the light emission for the light output.^40,46^ Additionally, the spectral linewidth as a function of injection current was also taken into account. On increasing the injection current in the range from 0.03 to 8.0 mA, Fig. 1(i) shows that the spectral linewidth exhibited a dramatic narrowing from 65 to 12 nm. When the injection current was beyond 8.0 mA, the spectral linewidth stabilized at a value of 10 nm.^44,45^ Therefore, by incorporating large-sized Ag nanoparticles, a single ZnO:Ga MW can be utilized to construct a heterojunction diode, with the NBE-type emission of ZnO:Ga dominating the optical output features.

**Fig. 2:**
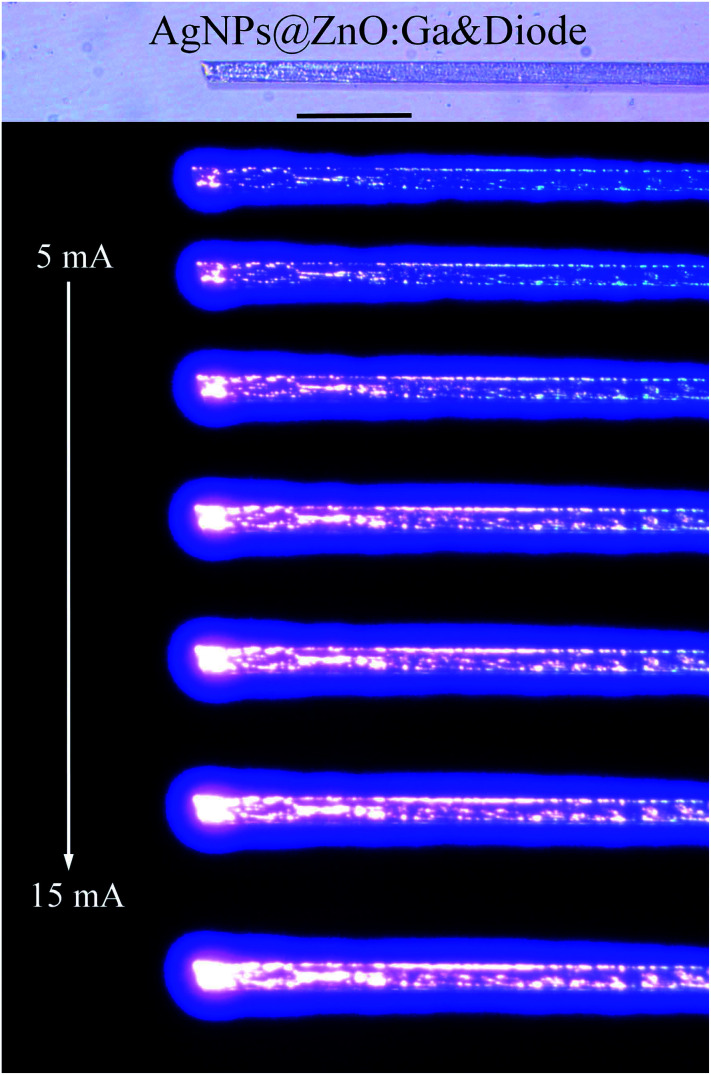
The optical microscopy images of ultraviolet light emission from the n-AgNPs@ZnO:Ga MW/p-GaN heterojunction diode, with the injection current ranging from 5 to 15 mA (the scale bar: 100 μm).

Optical characterization of the bright light emission from the single MW based heterojunction diode was recorded, as demonstrated in Fig. 2. The heterojunction diode began to emit blue-violet light once the injection current exceeded a certain value, and the brightness and emission regions increased with increasing injection current. Specifically, when the injection current was beyond a certain value, bright ultraviolet signals that dominated the EL emission were observed for the single AgNPs@ZnO:Ga MW based heterojunction diode. Additionally, the emitted ultraviolet light is so intense that it can be clearly observed by the naked eye in normal indoor lighting conditions.

**Fig. 3:**
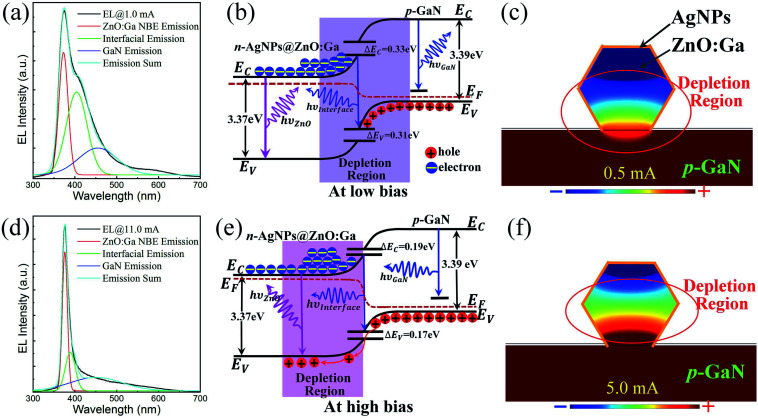
(a) Gaussian fitting of the emission spectrum, with the injection current of 1.0 mA. (b) Schematic diagram of the energy band structure of the AgNPs@ZnO:Ga/p-GaN heterojunction under low bias. (c) Electric potential field distribution along the cross-section of the heterojunction diode with the injection current of 0.5 mA. (d) Three distinct Gaussian deconvoluted sub-bands of the EL spectrum with the injection current of 11.0 mA, the dominant EL emission peak wavelength centered at 378.0 nm, and the spectral linewidth of 10.0 nm. (e) Schematic diagram of the energy band structure of the AgNPs@ZnO:Ga/p-GaN heterojunction under high bias. (f) Electric potential field distribution along the cross-section of the heterojunction diode with the forward injection current of 5.0 mA.

Taking one EL spectrum as an example (the injection current: 1.0 mA), compared with the PL spectrum of a single ZnO:Ga MW, the EL spectrum exhibited broadband emission characteristics with a spectral linewidth of 60 nm. The EL emission band extended asymmetrically from 350 to 500 nm. In addition to the NBE-type emission of a single ZnO:Ga MW, other emission should also be included, such as the interfacial emission from n-AgNPs@ZnO:Ga/p-GaN, and emission of the p-GaN layer.^2,6,40,47^ In the fabrication process, an energy barrier was formed at the heterostructure interface of the AgNPs@ZnO:Ga MW and p-GaN. When the diode is operated under low bias, the hole and electron distributions may be approximately equal at the interface. In consequence, the broadband EL emission from the single MW based heterojunction diode could be assigned to combined emission from the single AgNPs@ZnO:Ga MW, p-GaN and the AgNPs@ZnO:Ga/GaN interface.^6,23,40,45^Fig. 3(a) reveals that the EL spectrum was divided into three distinct subpeaks by the Gaussian function analysis, which were centered around 372.0 nm, 405.5 nm, and 455.6 nm. By comparing with the PL spectrum of a single ZnO:Ga MW, the light emission sub-bands centered around 372.0 and 455.6 nm were derived from typical NBE recombination in the ZnO:Ga MW, and electron transition from the conduction band to the deep Mg-related acceptor level in the p-GaN layer, respectively. The light emission band centered around 405.5 nm could be attributed to the interfacial recombination of the electrons in ZnO:Ga and the holes in p-GaN, and the energy barrier heights at the interface for the holes and the electrons were calculated to be about 0.31 eV and 0.33 eV, respectively, as illustrated in Fig. 3(b). Therefore, the broadband EL emission was ascribed to the superposition of the strong near-bandgap recombination of ZnO:Ga, the relatively weak interfacial radiation, and the much weaker emission from the p-GaN layer. Further, to better understand the underlying physical mechanisms of ultraviolet light emission from the electrically biased single MW based heterojunction diode, the electrostatic potential field distribution was investigated by numerical simulation. By means of the computational fluid dynamics approach, Fig. 3(c) shows that the depletion region was mainly distributed towards the interface between the AgNPs@ZnO:Ga MW and p-GaN under a low injection current of 0.5 mA.^2,40,47^

On increasing the injection current to 11.0 mA, the EL spectrum showed a dominant emission peak wavelength centered at 378.0 nm, accompanied by a reduction in the spectral linewidth to 10 nm. The EL spectrum was also decomposed into three subpeaks, as demonstrated in Fig. 3(d). It is discernable that the interfacial emission and the emission from the p-GaN layer were completely suppressed. Thus, the EL emission from the heterojunction diode was dominated by the strong near-bandgap recombination of the AgNPs@ZnO:Ga MW. Further, to explore the principle of the typical NBE emission, the heterostructure band diagram for n-AgNPs@ZnO:Ga/p-GaN is depicted in Fig. 3(e). The barrier heights at the interface for holes and electrons were calculated to be 0.17 eV and 0.19 eV, respectively.^6,40,45^ As is well-known, by incorporating metal nanostructures, the electronic transport properties of single semiconducting MWs can be enhanced greatly. Following on, Ag nanoparticle deposition can also be employed to achieve effective carrier injection into the heterojunction diode. It is obvious that the carrier concentration confined in the single AgNPs@ZnO:Ga MW increased accordingly on increasing the injection current. Nevertheless, electron mobility would be decreased at higher forwards bias. The reduction of electron mobility could be associated with enhanced scattering caused by significantly increasing the forwards bias.^41,48,49^ Therefore, once the applied bias exceeds a certain value, the holes in the p-GaN layer can tunnel through the barrier and be injected into the MW, while electrons will be restrained and accumulated in the MW due to the reduction of electron mobility. Finally, higher efficiency excitonic radiative recombination can occur in the AgNPs@ZnO:Ga MW active region.^18,44,50^ In addition, the electrostatic potential field distribution was investigated, as shown in Fig. 3(f). Under high injection current, such as 5.0 mA, it is obvious that the depletion region is mainly confined in the AgNPs@ZnO:Ga MW. Consequently, the incorporation of large-sized Ag nanoparticles deposited on the MW can lead to confinement of the carriers in the n-type ZnO:Ga MW, resulting in enhancement of the NBE-type emission of the single MW based heterojunction diode, accompanied by effective suppression of the interfacial emission.

**Fig. 4:**
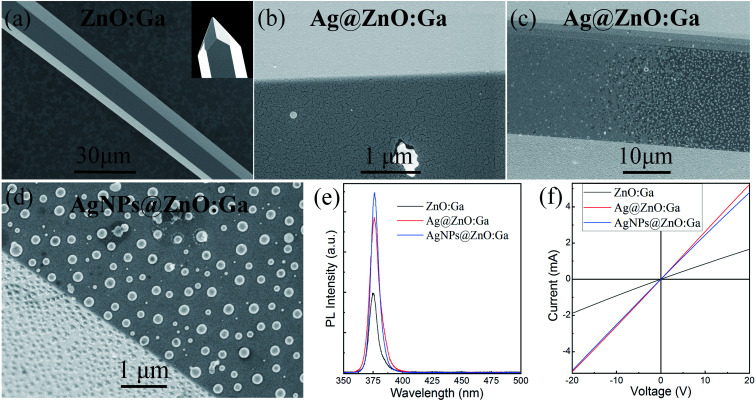
(a) SEM image of a single ZnO:Ga MW, with the tip of the pencil-like ZnO:Ga MW demonstrated in the inset. (b) SEM image of a single ZnO:Ga MW with Ag quasiparticle nanofilm deposition. (c) SEM image of a single MW in the critical region between emissive and non-emissive, indicating the transition region in which Ag quasiparticle nanofilms are transformed into isolated Ag nanoparticles. (d) SEM image of Ag nanoparticles deposited on the MW. (e) PL emission spectra of a single ZnO:Ga MW, a ZnO:Ga MW with Ag quasiparticle nanofilm decoration (the sputtering time: 300 s), and a ZnO:Ga MW covered by isolated Ag nanoparticles. (f) The *I*–*V* characteristics of a single ZnO:Ga MW, a ZnO:Ga MW with Ag quasiparticle nanofilm decoration (the sputtering time: 300 s), and a ZnO:Ga MW covered by isolated Ag nanoparticles.

As mentioned in the Experimental section, individual ZnO:Ga MWs were synthesized *via* CVD methods. Fig. 4(a) shows an SEM image of an as-synthesized ZnO:Ga MW, with the tip of the pencil-like ZnO:Ga MW also displayed in the inset. One can see that the MW was synthesized directly on the target with a diameter of ∼20 μm. The as-synthesized MWs have smooth facets and a regular hexagonal cross-section. A corundum boat together with an appropriate growth temperature (≥1150 °C) should be employed to synthesize pencil-like MWs.^38,40,51^ A SEM image of a single MW on incorporating Ag quasiparticle nanofilms (the sputtering time: 300 s) is shown in Fig. 4(b). Thus, Ag quasiparticle nanofilms were deposited on the MWs. Thus, a single Ag@ZnO:Ga MW based fluorescent filament light source was fabricated. When the applied bias was beyond a certain value, incandescent-type emission was observed, with the emission regions located towards the center. The segments of the MWs located in the incandescent-type emission regions were characterized. Fig. 4(c) illustrates the transition region in which Ag quasiparticle nanofilms were transformed from quasiparticle nanofilms into isolated Ag nanoparticles in the critical regions between emissive and non-emissive.^24,38^ In the incandescent-type emission regions, physically isolated Ag nanoparticles with random distribution were fabricated, as displayed in Fig. 4(d). It also reveals that the average diameter of the nanoparticles was about 200 nm.

To explore the influence of the deposited Ag nanostructures on the optical properties of the as-synthesized ZnO:Ga MWs, optical measurements were carried out using a micro-photoluminescence (μ-PL) system, with a He–Cd laser of 325 nm wavelength serving as the excitation light source. The excited laser beam was focused on the MWs through a microscope objective, and the μ-PL signals were detected by the same objective and recorded by a CCD detector. Fig. 4(e) shows the room temperature μ-PL spectra of the MWs. The μ-PL spectrum of a single ZnO:Ga MW demonstrated a dominant emission peak wavelength centered at 376.0 nm, accompanied by negligible broad emission in the visible spectral band. On incorporating Ag quasiparticle nanofilms, enhancement of the NBE-type emission was achieved. When the deposited Ag quasiparticle nanofilms were annealed into large-sized Ag nanoparticles, the NBE-type emission of the ZnO:Ga MW could be further improved. Additionally, the influence of the deposited Ag nanostructures on the electrical properties of a single ZnO:Ga MW was also taken into account. The *I*–*V* characteristics showed that the electronic transport behavior of the single ZnO:Ga MW was also enhanced greatly, as indicated in Fig. 4(f). Therefore, not only the Ag quasiparticle nanofilms, but also the Joule heating effect which induced the aggregation of isolated Ag nanoparticles deposited on the as-synthesized ZnO:Ga MW, can be employed to modulate the optical and electronic transport properties of the single wire.^23,24,38^

**Fig. 5:**
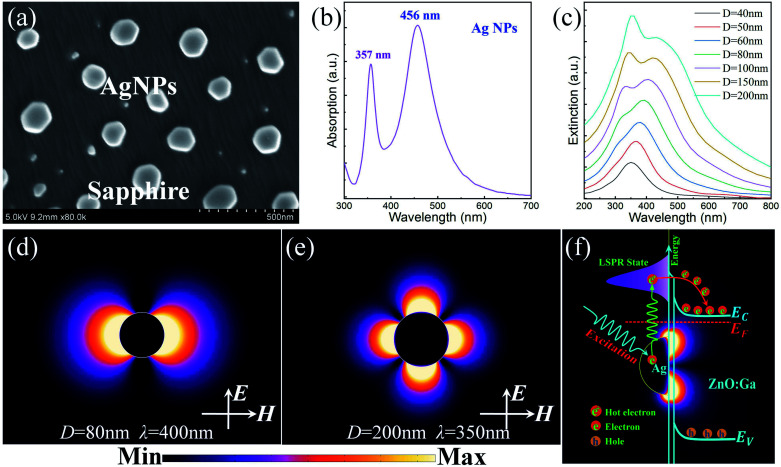
(a) SEM image of Ag nanoparticles prepared on sapphire, the average diameter *d* ∼ 200 nm. (b) Absorption spectrum of Ag nanoparticles with two discrete absorption peaks centered around 357 and 456 nm. (c) The calculated extinction spectra of Ag nanoparticles with diameters ranging from 40 to 200 nm. (d) The spatial distribution of the electric field intensity for a Ag nanoparticle with diameter *D* = 80 nm, resonant wavelength *λ* = 400 nm. (e) The spatial distribution of the electric field intensity for a Ag nanoparticle with diameter *D* = 200 nm, resonant wavelength *λ* = 350 nm. (f) A schematic diagram of non-radiative decay of hybrid quadrupole plasmons, inducing the generation of hot electrons, which are then injected into the neighboring ZnO:Ga MW.

Metal plasmons, the collective oscillation of electrons arising from electronic processes towards the surface of metal nanostructures, have long been the subject of intense research interest. The electric field enhancement and localization can be employed for versatile applications, such as plasmon enhanced Raman scattering, light emission enhancement, nonlinear optical processes, plasmon enhanced high-performance photodetection, and so on.^19,20,52–54^ To address the plasmonic characteristics of the large-sized Ag nanoparticles deposited on the ZnO:Ga MW, Ag nanoparticles with identical sizes were also prepared on sapphire, as shown in Fig. 5(a). The absorption spectrum of the Ag nanoparticles was obtained by using ultraviolet-visible spectroscopy in absorbance mode. It showed two different absorption peaks centered at 357 nm and 456 nm, as indicated in Fig. 5(b). The absorption peak centered at 357 nm was attributed to hybrid quadrupole plasmons, while the other absorption peak centered at 456 nm could be assigned to dipole plasmons.^25,34,38^ Theoretical calculations and simulations were carried out based on the standard Mie theory for spherical particles and compared with the experimental results.^25–27^Fig. 5(c) demonstrates that when a small spherical metallic nanoparticle (*D* ≤ 80 nm) was irradiated by the incident light, the collective oscillation of the conduction electrons, called the dipole plasmons, could be excited. On increasing the size, the dipole plasmon band became broadened, which mainly resulted from radiative losses. That is, the plasmonic mode cannot be trapped in the particle and it couples to the propagating light in free space, resulting in a gradual broadening that increases with the coupling strength. When the diameter exceeded 100 nm, another weak absorption peak appeared in the shorter wavelength spectral region. On further increasing the diameter of the particle, higher order resonance modes, especially the quadrupole mode, became important in the extinction spectra and occupied a dominant position. In a contrast, the intensity of the dipole resonance peak was gradually decreased and broadened, accompanied by a significant red shift. These results indicate that high-order plasmonic modes could occur in isolated nanoparticles with relatively large size due to the inhomogeneous distribution of electron density. Meanwhile, this implied that large-sized nanoparticles interacted with radiation light to a much greater extent than small nanoparticles because of the larger number of electrons participating in the plasmon resonances.^25,27,29,35^

To ascertain the plasmonic modes of the deposited Ag nanoparticles, the distribution of the integrated electromagnetic field |*E*|^2^ intensity was carried out by using the finite difference time-domain (FDTD) method. Taking a Ag nanoparticle with a diameter of 80 nm for instance, the calculated electromagnetic field |*E*|^2^ intensity distribution showed that the plasmonic resonance had a dipolar nature for the long-wavelength absorption peak centered around 400 nm, as shown in Fig. 5(d). As a comparison, the electromagnetic field |*E*|^2^ intensity of a Ag nanoparticle with a particle diameter of 200 nm is depicted in Fig. 5(e). It showed plasmonic quadrupole mode characteristics in the short-wavelength spectral band.^25–27,29^ As previously reported, increasing the size of Ag nanoparticles can lead to a linear red shift of the plasmonic dipolar resonance modes, as well as a decrease in the interparticle gap spacing. Meanwhile, the plasmonic quadrupole resonance modes of the Ag nanoparticle aggregates remained unchanged, with the dominant absorption peak centered at 357 nm. The absorption peak remaining unchanged may be attributed to the shorter decay length of the quadrupolar field in comparison with that of the plasmonic dipole mode. Thereby, the absorption peak of the quadrupolar resonance was not significantly affected by the adjacent nanoparticles. As a result, hybrid quadrupole plasmons can occur in the deposited Ag nanoparticles.^25,27,35^ On adjusting the sputtering time, the dominant absorption wavelengths of the quadrupole plasmons remained practically unchanged, while the other dipolar mode red shifted toward a longer wavelength spectral band.

Obviously, the PL emission of a single ZnO:Ga MW cannot match the absorption peaks of the Ag nanoparticle aggregates, whether for quadrupole plasmons or dipole plasmons. Thus, the PL emission enhancement of a single ZnO:Ga MW with Ag nanoparticle decoration cannot be attributed to resonant coupling between ZnO:Ga excitons and AgNPs plasmons at the interface.^21,22,55^ When the output radiation from the heterojunction LED is absorbed by the deposited Ag nanoparticles, hybrid quadrupole plasmons can be excited. After excitation, nonradiative decay can occur through intraband excitations within the conduction band or through interband excitations, leading to transitions between the conduction band and other bands. Moreover, when they possess high enough energy, the energized electrons can overcome the metal–semiconductor contact barrier and be injected into the conduction band of the neighboring ZnO:Ga.^56–60^ Further, to interpret the plasmon mediated generation, injection, and collection of energized electrons in AgNPs@ZnO:Ga MW based heterostructured light-emitting devices, a schematic diagram of hybrid quadrupole plasmons inducing the hot electron generation and injection procedure at the AgNPs–ZnO:Ga interface is depicted in Fig. 5(f). In the deposited Ag nanoparticles, plasmons induced the generation of hot electrons which could be injected into the neighboring ZnO:Ga MW. Therefore, the single ZnO:Ga MW can be employed as a passive component for charge collection.

**Fig. 6:**
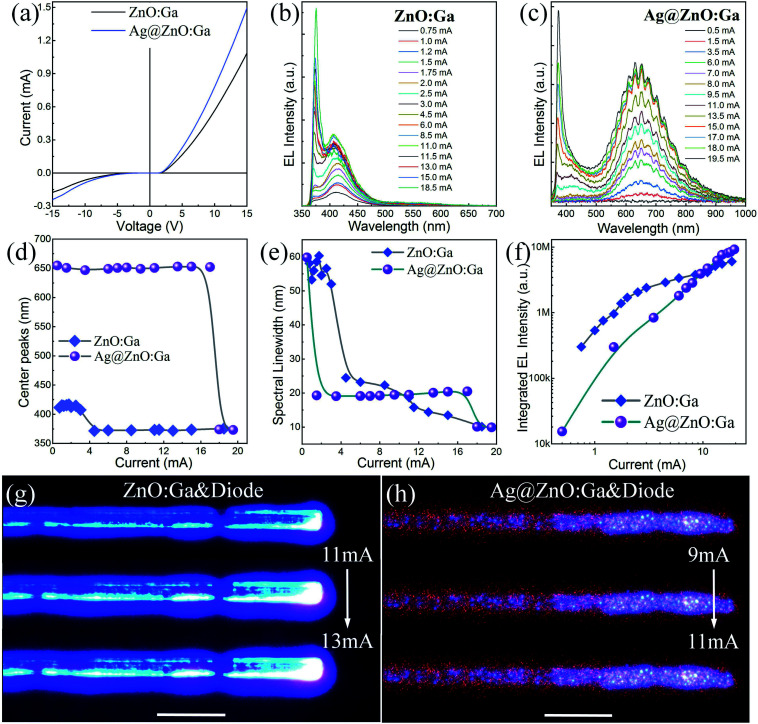
EL emission characteristics of the single MW based heterojunction LEDs: (a) *I*–*V* curves illustrating the rectifying behavior of single MW based heterojunction diodes. (b) EL spectra of a single ZnO:Ga MW based heterojunction diode, with the injection current ranging from 0.75 to 18.5 mA. (c) EL spectra of a single Ag@ZnO:Ga MW based heterojunction LED (the sputtering time: 300 s), with the injection current ranging from 0.5 to 19.5 mA. (d) The dominant emission peak wavelengths of the heterojunction LEDs as a function of the injection current. (e) Spectral linewidth *versus* the injection current. (f) Integrated EL emission intensity of the heterojunction LEDs as a function of the injection current. (g) The optical microscopy images of the blue-ultraviolet light emission from the n-ZnO:Ga MW/p-GaN heterojunction diode. (h) The optical microscopy images of the red-ultraviolet light emission from the n-Ag@ZnO:Ga MW/p-GaN heterojunction diode.

To ensure that the hybrid quadrupole plasmon induced generation and injection of hot electrons is responsible for the predominant NBE-type emission from the single MW based heterojunction diode, another single ZnO:Ga MW prepared *via* Ag quasiparticle nanofilm decoration was also employed to construct heterojunction diodes. Compared with the single bare ZnO:Ga MW based heterojunction diode, Fig. 6(a) indicates that the *I*–*V* rectifying characteristics were enhanced by introducing Ag quasiparticle nanofilm decoration. The typical EL spectra of the single ZnO:Ga MW based heterojunction diode were recorded under a forward injection current ranging from 0.75 to 18.5 mA, as displayed in Fig. 6(b). In addition to the ultraviolet emission centered around 375.0 nm, another broadband visible emission from 400.0 nm to 475.0 nm with central peak wavelength centered around 415.0 nm was also observed. As we mentioned above, the ultraviolet light emission can be assigned to NBE-type emission, while the broadband emission centered around 415.0 nm can be attributed to interfacial emission in the n-ZnO:Ga/p-GaN contact region.^2,40,45^ Under low injection current ranging from 0.75 to 3.0 mA, the dominant emission peak wavelength was centered around 415.0 nm in the blue spectral band covering the range from 375.0 to 475.0 nm. On further increasing the injection current from 4.5 to 18.5 mA, the ultraviolet emission increased significantly, and then dominated the EL spectrum.^6,40^

On introducing Ag quasiparticle nanofilm decoration (the sputtering time: 300 s), the EL spectra of the single Ag@ZnO:Ga MW based heterojunction diode were also collected, as shown in Fig. 6(c). Under low injection current ranging from 0.5 to 11.0 mA, the dominant emission peak wavelength was centered around 650.0 nm, accompanied by relatively weaker emission centered around 372.0 nm in the ultraviolet spectral region. By contrast, the PL emission of the single ZnO:Ga MW showed that the dominant ultraviolet emission centered around 375.0 nm was attributed to the radiative recombination of ZnO:Ga excitons; while the other negligible visible emission was derived from Ga impurity related radiative transitions.^39,41,61^ Additionally, on incorporating Ag quasiparticle nanofilms, the electronic transport properties of the single ZnO:Ga MW were greatly enhanced.^23,38^ Under low forward injection currents ranging from 0.5 to 11.0 mA, electrons from the Ga impurity related level of ZnO:Ga arrived at the n-ZnO:Ga/p-GaN interface, and would then be captured by the holes of the Mg-related deep acceptor levels of the p-GaN layer, yielding a broad red light band around 650.0 nm.^62,63^ On further increasing the injection current from 6.0 to 15.0 mA, ultraviolet light emission centered around 370.0 nm was observed, but the EL spectra were still dominated by the red emission. When the injection current reached beyond 15.0 mA, a sharp increase in the ultraviolet emission appeared. Thereby, the typical NBE emission of the single Ag@ZnO:Ga MW based heterojunction diode increased dramatically, and finally became prominent in the EL spectra. Meanwhile, the red EL emission centered at 650.0 nm was effectively suppressed. The limited increase in the broad red emission may be ascribed to the lower hole concentration in the p-GaN layer.^48,63^ In addition, the ultraviolet emission showed a dominant peak wavelength centered around 375.0 nm, together with a narrowing of the spectral linewidth of the sub-band peaks to around 10 nm. The ultraviolet emission of the single MW based heterojunction diode can be assigned to NBE-type emission of ZnO:Ga. That is, holes in the p-GaN layer could tunnel into the ZnO:Ga MW at the interface of the emission device. Therefore, NBE-type ultraviolet emission could still be achieved by a single MW based heterojunction by introducing Ag quasiparticle nanofilm deposition. Regrettably, the EL spectra are not dominated by the NBE-type emission of the single Ag@ZnO:Ga MW.

To gain more insight into the light-emitting behavior, the dominant emission peak wavelengths of the single MW based heterojunction diodes as a function of the injection current are depicted in Fig. 6(d). Compared with the bare ZnO:Ga MW based heterojunction diode, the introduction of Ag quasiparticle nanofilms cannot be utilized to achieve a high-performance ultraviolet light source, that is, the EL spectra of the single MW based heterojunction diode are not dominated by the NBE-type emission of ZnO:Ga MWs. Meanwhile, Fig. 6(e) presents the relationship between the spectral linewidth and the injection current. It is clear that significant shifts of the emission line shapes in the ultraviolet spectral region were observed for the heterojunction LEDs, whether or not they incorporated Ag quasiparticle nanofilms. Fig. 6(f) displays the integrated EL emission intensity as a function of the injection current. The introduction of the Ag quasiparticle nanofilm covering resulted in lower luminous efficiency and luminous intensity of the single MW based heterojunction diodes.^62,64^ Besides, optical characterization of the light emission from the heterojunction diodes was carried out. Optical microscopy images of the bright blue-violet light emission were obtained for the single bare ZnO:Ga MW based heterojunction diode, as illustrated in Fig. 6(g). On increasing the injection current, the emission behavior also indicated that a significant transition of the emission from blue to violet can be achieved. On incorporating Ag quasiparticle nanofilms, optical microscopy images of the bright red light accompanied by violet light were recorded for the single Ag@ZnO:Ga MW based heterojunction diode, as shown in Fig. 6(h).

Ag quasiparticle nanofilms were also deposited on a ZnO:Ga MW with the sputtering time of 200 s. When the applied bias exceeded a certain value, bright incandescent-type emission was observed. Physically isolated Ag nanoparticles with average diameters *d* ∼ 100 nm located in the emission region were formed. The corresponding SEM images were obtained and are shown in Fig. S1 in the ESI.† Afterwards, the segment of the ZnO:Ga MW covered by Ag nanoparticles was also employed to construct a heterojunction diode. Optical characterization of the light emission and the emitted photons was carried out. The EL emission characteristics are illustrated in Fig. S2 in the ESI.† Compared with the single bare ZnO:Ga MW based heterojunction diode, EL emission enhancement was realized. The enhancement could be ascribed to near-field enhancement through efficient coupling between excitons of the ZnO:Ga MW and dipole plasmons of the deposited Ag nanoparticles. Unfortunately, in addition to the emission enhancement, the EL emission spectra demonstrated that typical ultraviolet emission peaks centered around 375.0 nm were observed, which were attributed to the NBE emission of the single ZnO:Ga MW. The spectral linewidths of the EL emission spectra were much wider than that of the PL spectrum of the single MW.^18,22,34,65^ The detailed EL emission characteristics can be seen in Fig. S3 in the ESI.† In general, the incorporation of large-sized Ag nanoparticles can be utilized to achieve NBE-type emission, which dominates the EL emission of single ZnO:Ga MW based heterojunction diodes.

## 4 Conclusion

In summary, by employing a single AgNPs@ZnO:Ga MW as an active layer, heterojunction diodes with strong, stable, and spectrally pure ultraviolet emission were fabricated. The dominant emission peak wavelength was centered around 378.0 nm, accompanied by a reduction in the spectral linewidth to around 10 nm. The NBE-type emission dominated EL spectra were explained by hybrid quadrupole plasmons inducing the generation of hot electrons, which were injected into the conduction band of the adjacent ZnO:Ga MW. The excitation of the hybrid quadrupole plasmons and the subsequent nonradiative decay were investigated in detail. By taking advantage of the large-sized Ag nanoparticles, this work also presented deep insights into manipulating high-order plasmonic oscillations, which have potential in the construction of a lower-cost effective alternative to traditional ultraviolet light-emitting devices and diodes. Furthermore, the fabrication of individual ZnO:Ga MWs covered by isolated metallic nanoparticles with controlled sizes and gap distances opens new avenues for the design of novel optoelectronic devices with high performance and high efficiency.

## Conflicts of interest

There are no conflicts to declare.

## Supplementary Material

NA-002-C9NA00777F-s001
