# Supplementary material for: Hybrid quadrupole plasmon induced spectrally pure ultraviolet emission from a single AgNPs@ZnO:Ga microwire based heterojunction diode
Source: Nanoscale Adv. 2020 Feb 24;2(3):1340–51. doi: 10.1039/c9na00777f (PMC9417069; doi:10.1039/c9na00777f)
Supplement: NA-002-C9NA00777F-s001 [file NA-002-C9NA00777F-s001.pdf]

Electronic Supplementary Material (ESI) for Nanoscale Advances.
This journal is © The Royal Society of Chemistry 2020

Supporting Information

Hybrid Quadrupole Plasmons Induced Spectrally Pure Ultraviolet Emission from Single AgNPs@ZnO:Ga Microwire based Heterojunction Diode

Xiangbo Zhou,[a] Mingming Jiang,[*,a,b] Yuting Wu,[a] Kunjie Ma,[a] Yang Liu,[a] Peng Wan,[a] Caixia Kan,[*,a,b] and Daning Shi[*,a]

[a] *College of Science, Nanjing University of Aeronautics and Astronautics, No. 29 Jiangjun Road, Nanjing 210016, China*

[b] *Key Laboratory for Intelligent Nano Materials and Devices (MOE), Nanjing University of Aeronautics and Astronautics, Nanjing 210016, China.*

*Corresponding Authors:

Prof. Mingming Jiang: mmjiang@nuaa.edu.cn

Prof. Caixia Kan: cxkan@@nuaa.edu.cn

Prof. Daning Shi: shi@nuaa.edu.cn.

1. Single ZnO:Ga MW prepared with small-sized Ag nanoparticles decoration (*d*~100 nm)

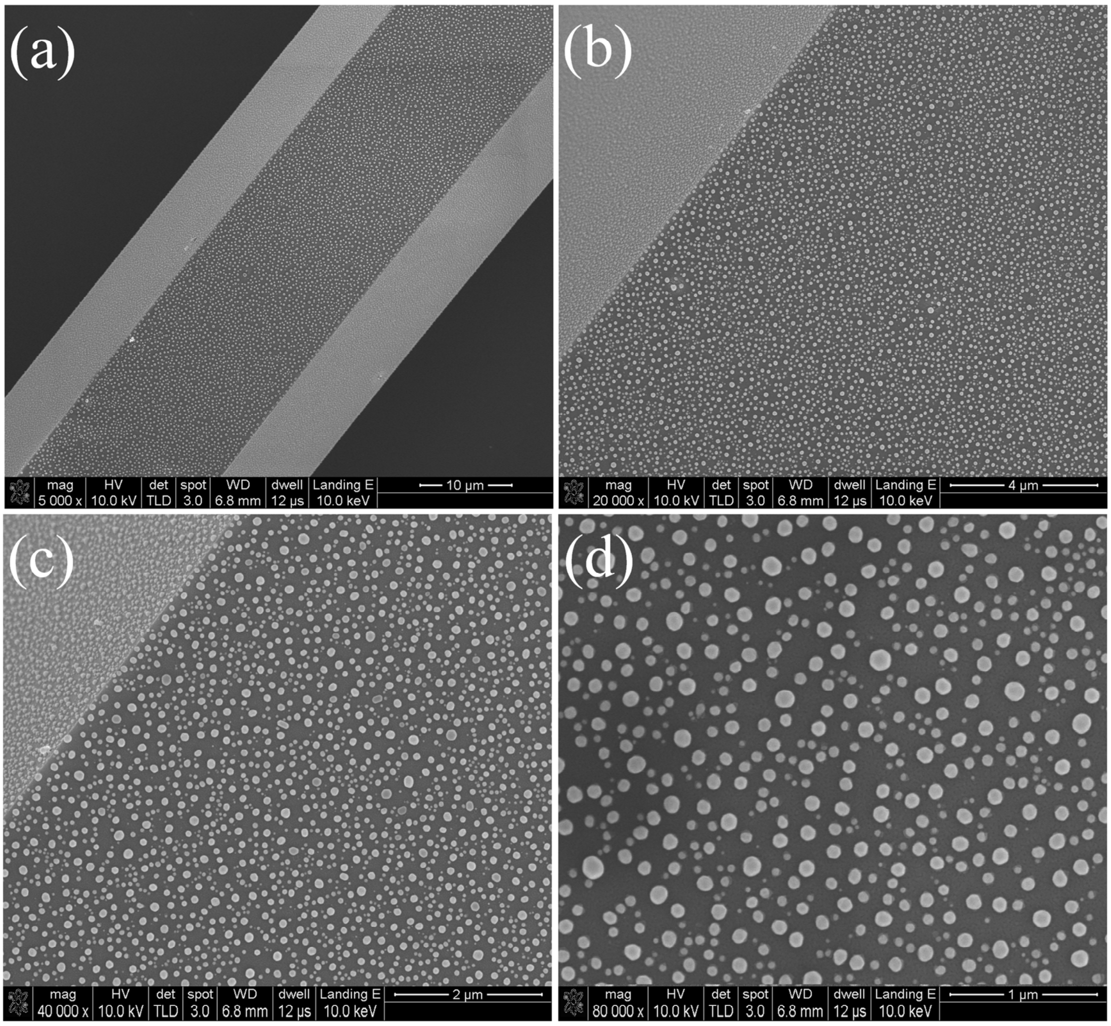

Figure S1: SEM images of single ZnO:Ga MW prepared with small-sized Ag nanoparticles decoration: (a) The segment of ZnO:Ga MW covered by Ag nanoparticles, which located in the incandescent-type lighting region. (b)-(d) Amplified SEM images of the Ag nanoparticles deposited on the MW.

A single ZnO:Ga MW was selected to construct fluorescent filament light source. When the applied bias exceeded a certain value, bright visible incandescent-type lighting was observed, with the emission region located towards the center of the wire. Follow upon, Ag quasiparticle nanofilms with the sputtering time 200 s was also deposited on the ZnO:Ga MW. Still, bright incandescent-type emission was also captured once the applied bias beyond a certain value. Interestingly, physically isolated

Ag nanoparticles with average diameters d ~ 100 nm can be formed through Joule heating effect induced annealing procedure. The single ZnO:Ga MW covered by Ag nanoparticles (AgNPs@ZnO:Ga) was characterized in Figure S1.

2. EL emission features from single MW based heterojunction diodes

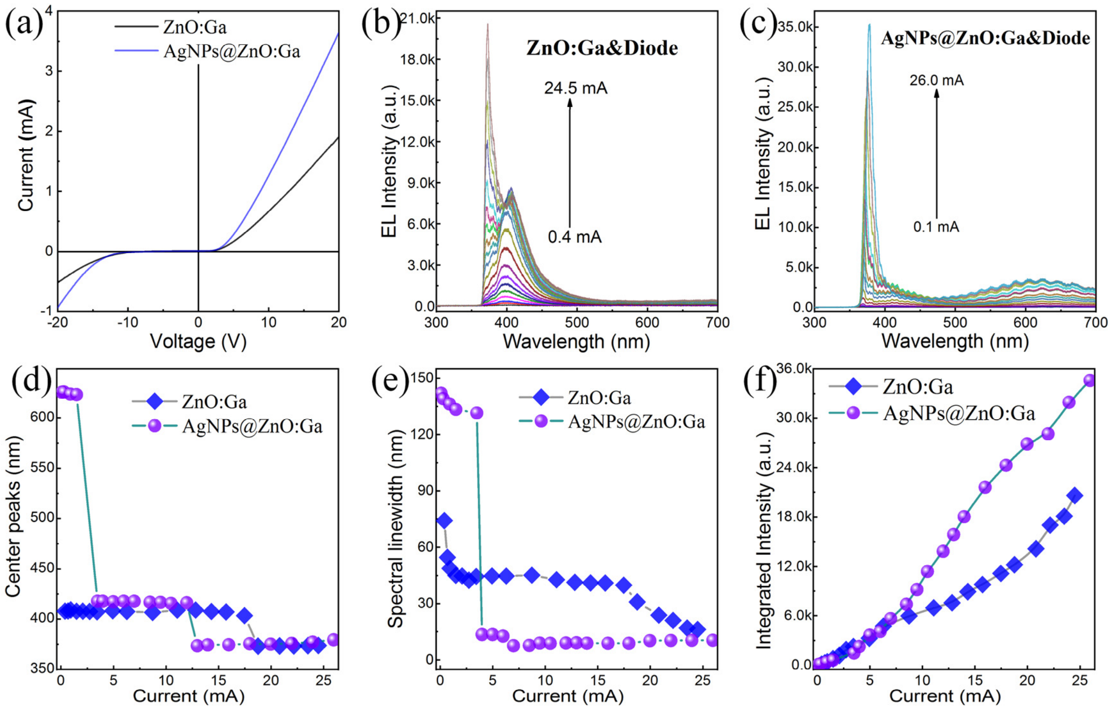

Figure S2: EL emission characteristics from single MW based heterojunction diodes: (a) I-V characteristics illustrated rectifying characteristic of single MW based heterostructured light-emitting devices. (b) EL emission spectra of single ZnO:Ga MW based heterojunction diode, with the injection current ranging from 0.4 to 24.5 mA. (c) EL emission spectra of single AgNPs@ZnO:Ga MW based heterojunction diode, with the injection current ranging from 0.1 mA to 26.0 mA. (d) The dominating emission peaks of the single MW based heterojunction diodes as a function of the injection current. (e) Spectral linewidth versus the injection current. (f) Integrated EL intensities of EL emissions as a function of the injection current.

The segment of ingle ZnO:Ga MW covered by small-sized Ag nanoparticles was also employed to construct heterojunction diodes. By comparison, the incorporating Ag nanoparticles can improve the carrier transport properties, as indicated in Fig. S2(a). The emitted photons were also collected. Figure S2(b) demonstrated EL emission spectra of single bare ZnO:Ga MW based heterojunction diode. At lower injection current, the dominating emission peaks centered around 405 nm. While increasing the

injection current, the dominated peaks was tuned to be 373.5 nm. By incorporating small-sized Ag nanoparticles, the EL emissions were dominated by the peaks centered around 375 nm. Unfortunately, another weaker emissions were also recorded in the visible band. To illustrate the influence of Ag nanoparticles on the EL emission characteristics of the single MW based heterojunction diode, the central emission peaks versus the injection current were depicted in Fig. S2(d). Figure S2(e) displayed the spectral linewidth as a function of the injection current. It indicated that the incorporation of Ag nanoparticles can lead to the narrower of the spectral linewidth of the emission diodes. Finally, the integrated emission intensity was calculated as a function of the injection current, as shown in Fig. S2(f). In a contrast, all the integrated EL emission intensities were considerably enhanced under various injection currents. By comparing with large-sized Ag nanoparticles deposition, the modulation of dipole plasmons on the EL emission characteristics was limited.

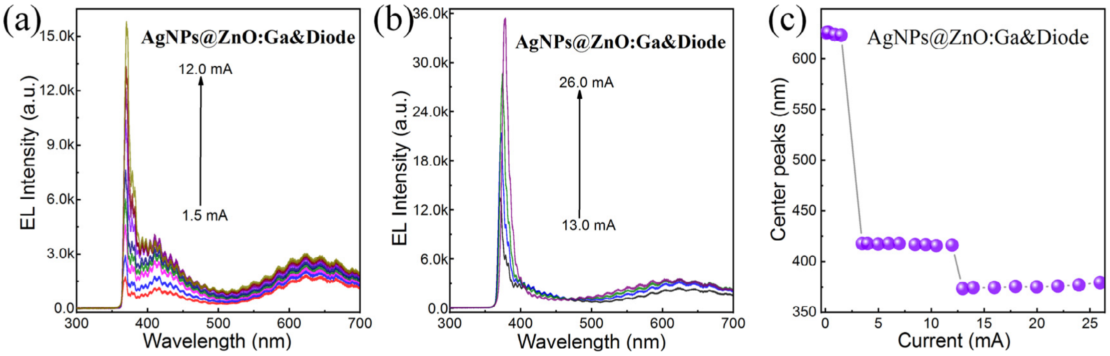

Figure S3: EL emission characteristics from the single AgNPs@ZnO:Ga MW based heterojunction diode: (a) EL emission spectra of single ZnO:Ga MW based heterojunction diode, with the injection current ranging from 1.5 to 12 mA. (b) EL emission spectra of single ZnO:Ga MW based heterojunction diode, with the injection current ranging from 13.0 to 26 mA. (c) The dominating emission peaks of the single MW based heterojunction diodes as a function of the injection current.

To gain more insight into the single AgNPs@ZnO:Ga MW based heterojunction diode light-emitting behaviors (the sputtering time: 200 s, *d*~100 nm), the dominating emission peaks of the single MW based heterojunction diodes as a function of the injection current were depicted in Figure S3. Under low injection current ranging from 0.1 to 1.5 mA, the dominant emissions centered around 640 nm, accompanied with a

relatively weaker emissions centered around 416 nm in the blue spectral band. Further to increase the injection current ranging from 1.5 to 12.0 mA, the ultraviolet emission increased significantly, and then would be dominated the EL emission spectra. But it is unfortunate that the interfacial emission and red emissions cannot be completely suppressed. Further to increase the injection currents ranging from 13.0 to 26.0 mA, the interfacial emissions was completely suppressed. Nevertheless, the broadband visible emission were still recorded.
